# Supplementary material for: An effluent pump family distributed across plant commensal bacteria conditions host- and organ-specific glucosinolate detoxification
Source: Nat Commun. 2025 Jul 1;16:5699. doi: 10.1038/s41467-025-61266-3 (PMC12215611; doi:10.1038/s41467-025-61266-3)
Supplement: Supplementary file 1 — Supplementary Information [file 41467_2025_61266_MOESM1_ESM.pdf]

Supplementary Figures for:

An effluent pump family distributed across plant commensal bacteria conditions host- and organ-specific glucosinolate detoxification

Dor Russ[1,2], Connor R. Fitzpatrick[1,2], Chinmay Saha[1,2], Theresa F. Law[1,2], Corbin D. Jones[1,3,4], Daniel J. Kliebenstein[5], Jeffery L. Dangl[1,2,*]

[1] Department of Biology, University of North Carolina at Chapel Hill, USA, 27599

[2] Howard Hughes Medical Institute, University of North Carolina at Chapel Hill, USA, 27599

[3] Integrative Program for Biological and Genome Sciences, University of North Carolina at Chapel Hill, USA, 27599

[4] Department of Genetics, University of North Carolina at Chapel Hill, USA, 27599

[5] Department of Plant Sciences, University of California Davis, Davis, CA, USA

[*] Corresponding author dangl@email.unc.edu

Supplementary Figure 1. The abundance of Paraburkholderia in wild soil

a

b

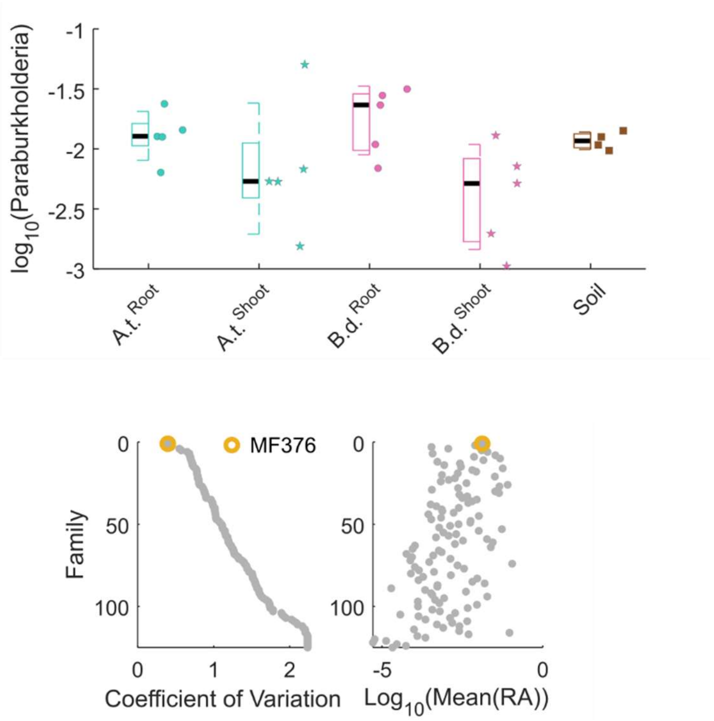

a, The Log transformed relative abundance of all members of the Paraburkholderia genus in the roots and shoots of both *Arabidopsis thaliana* Col-0 (A.t., cyan, 5 independent plants) and *Brachypodium distachyon* BD-21 (B.d., magenta, 5 independent plants) as well as in the soil (Brown, 4 independent samples) as calculated based on read counts of 16S rRNA gene amplicon sequencing. Paraburkholderia are detectable and maintain similar levels in all sample types and are stable across most samples. **b,** The coefficient of variation (CV, left) and log transformed mean relative abundance (right) of all bacterial families found in wild soil sorted by their CV. Paraburkholderia is the least noisy family (as measured by CV) across habitats and is the 15th ranked family in mean relative abundance.

Supplementary Figure 2. TnBarSeq results for *Paraburkholderia bryophila* MF376

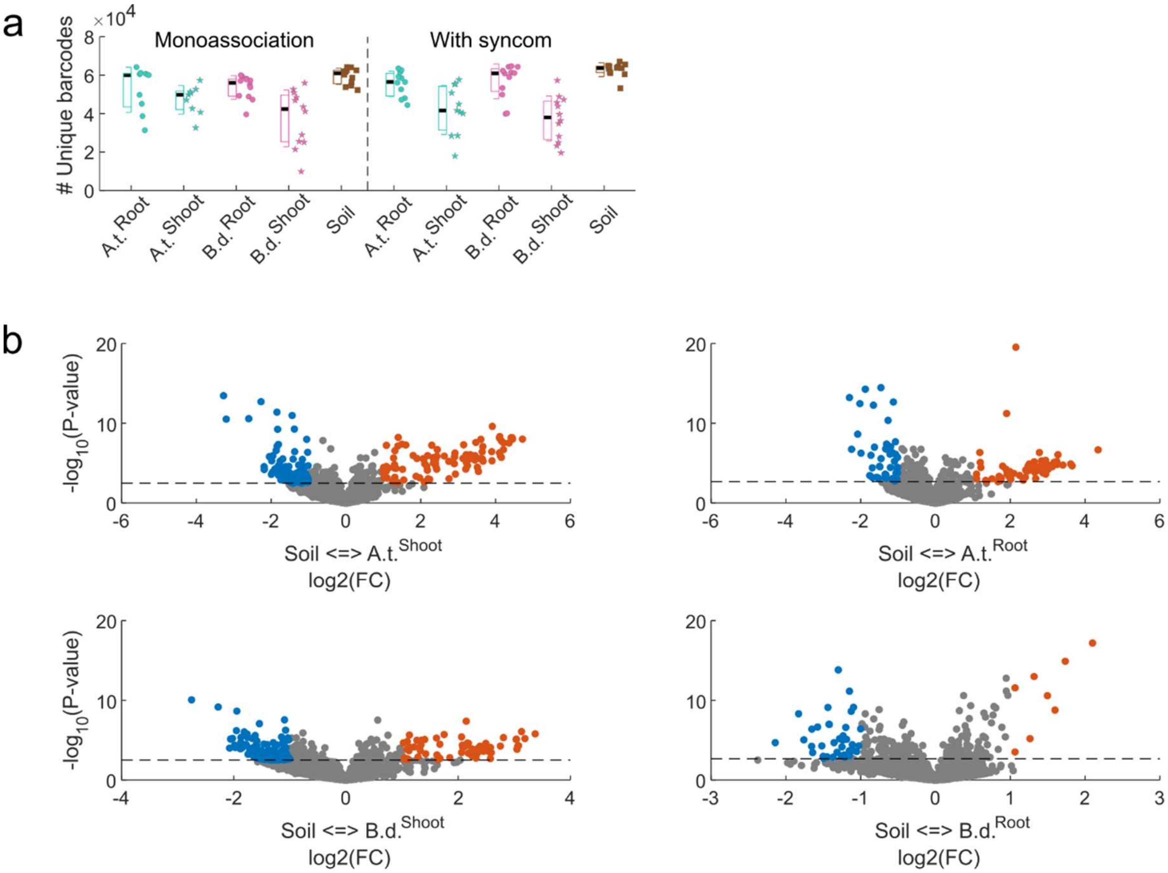

c

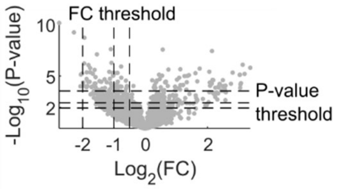

Total number of association genes

		Threshold Log_2(FC)		
		-0.5	-1	-2
Threshold P-value	0.01	635	372	16
	0.05	393	251	15
	0.1	196	133	15

Fraction of single organ genes

		Threshold Log_2(FC)		
		-0.5	-1	-2
Threshold P-value	0.01	0.77	0.8	0.81
	0.05	0.79	0.84	0.8
	0.1	0.82	0.83	0.8

a,The number of unique TnBarSeq barcodes identified for each biological sample. Boxplots show the median (black line), 1st and 3rd quartiles (box), and one standard deviation (whiskers) of each sample. Different sample types show similar numbers of unique barcodes. Adding the TnBarSeq library alone (Monoassociation, left) or with a small auxiliary community of four members (With syncom, right) had no effect on the number of unique barcodes. As we recovered barcodes from 5,962 MF376 genes and the average number of unique barcodes per sample is 50,544; each sample had an average of ~8.5 unique barcodes per gene. **b,** Volcano plot comparing the abundances of MF376 mutants in the soil (n=24 biologically independent samples) to their abundances in association with different plant tissues (n=24 and 20 biologically independent samples for B.d. and A.t. respectively). Fold changes (FC) and P-values for individual mutants were calculated based on soil samples and plant samples. Differentially abundant mutants that were

statistically different (significance threshold, FDR corrected linear model P-value < 0.05) are either depleted in association with plant tissue (plant association, blue, Log2(FC) < -1) or enriched in association with plant tissue (negative plant association, red, Log2(FC) > 1). All other mutants are defined as neutral (gray). **c,** The dominance of single organ association genes is robust across P-value and effect size thresholds. The identification of plant association genes relies on thresholds for both statistical significance (P-value) and biological significance (Fold Change, FC). To ensure that our observed organ and host specificity patterns were not threshold-dependent, we performed sensitivity analyses using varying cutoffs for statistical tests (FDR α) and fold change. While the total number of identified association genes varied substantially across thresholds (ranging from 15 to 661, left table), the proportion of single organ association genes remained remarkably consistent (0.77-0.83, right table), confirming the robustness of our findings.

Supplementary figure 3. TnBarSeq results from *Pseudomonas simiae* WCS417 recapitulates our findings using *Paraburkholderia bryophila* MF376

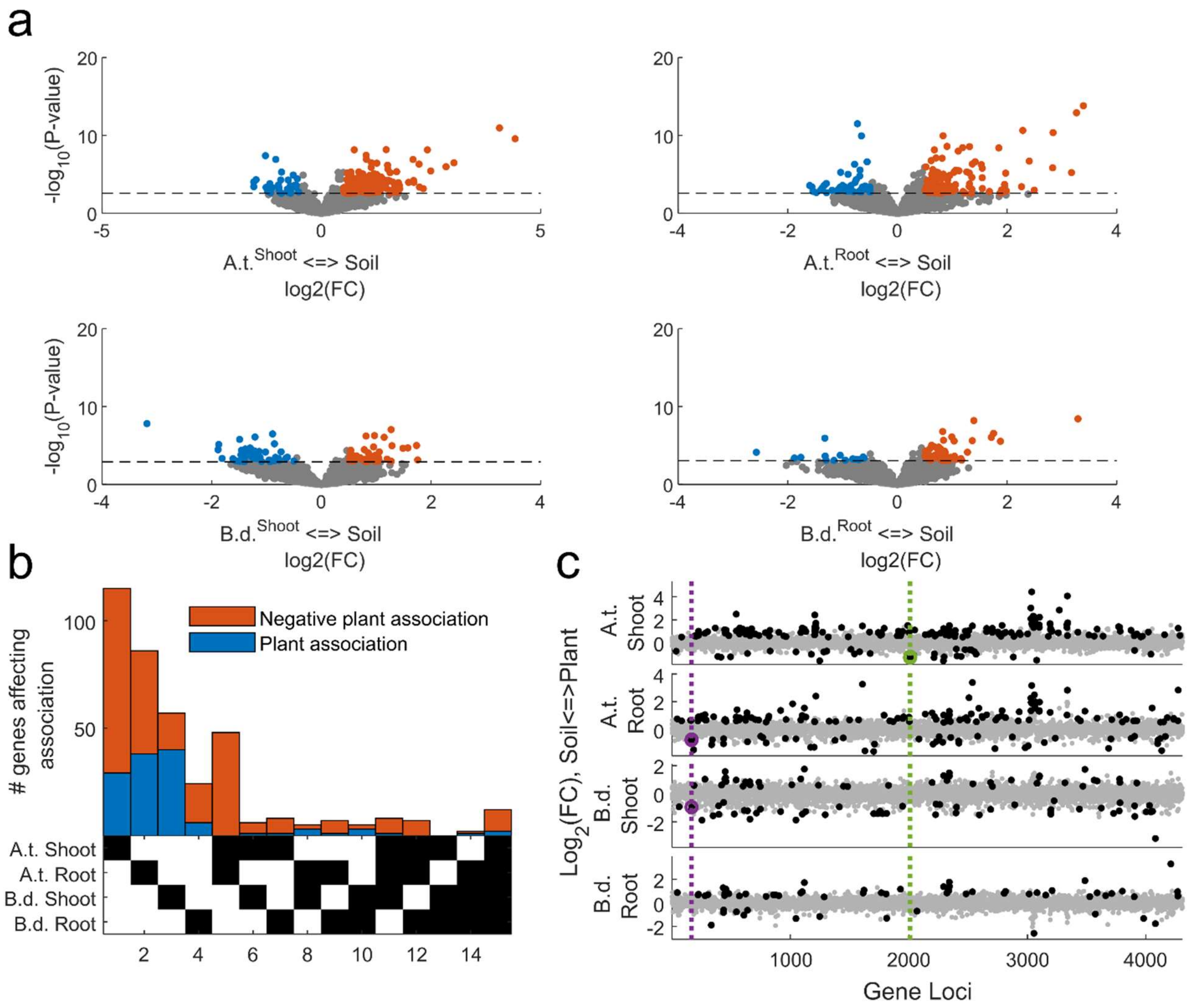

a, Volcano plot comparing the abundances of WCS417 mutants in the soil to their abundances in association with different plant tissue. Fold changes (FC) and P-values for individual mutants were calculated based on soil samples (n=24 biologically independent samples) and plant samples (n=24 and 22 biologically independent samples for B.d. and A.t. respectively). Differentially abundant mutants that were statistically different (significance threshold, FDR corrected linear model P-value < 0.05) are either depleted in association with plant tissue (plant association, blue, Log2(FC) < -0.5) or enriched in association with plant tissue (negative plant association, red, Log2(FC) > 0.5). All other mutants are defined as neutral (gray). As the recovery of WCS417 barcodes was not as successful as that of MF376 and only few genes satisfied the threshold of |Log2(FC)| > 1, we reduced the stringency of our criteria to |Log2(FC)| > 0.5. **b,** An upset plot of genes that affect plant association by WCS417. Genes that positively (blue) or negatively (red) affect association with different host and organ combinations (bottom, black squares). Full list of genes is in extended data table 3. Like Fig 2a (for MF376) most WCS417 plant association genes are highly specific to both host and organ. **c,** Locus position in the WCS417 genome of statistically significant mutants in plant association (black dots). Organ specific efflux pump systems benefit the

association with Arabidopsis shoots (green) or roots (purple) but not with other organs and host. Log2(FC) of mutant abundance in association with plant tissue compared to soil is plotted against their location on the genome. A single subunit of the root specific pump (a periplasmic gene) is also identified as a plant association gene in *Brachypodium distachyon* BD21 shoots.

Supplementary figure 4. Wildtype and d90 mutant *Paraburkholderia bryophila* MF376 grow to the same level in media without leaf extract.

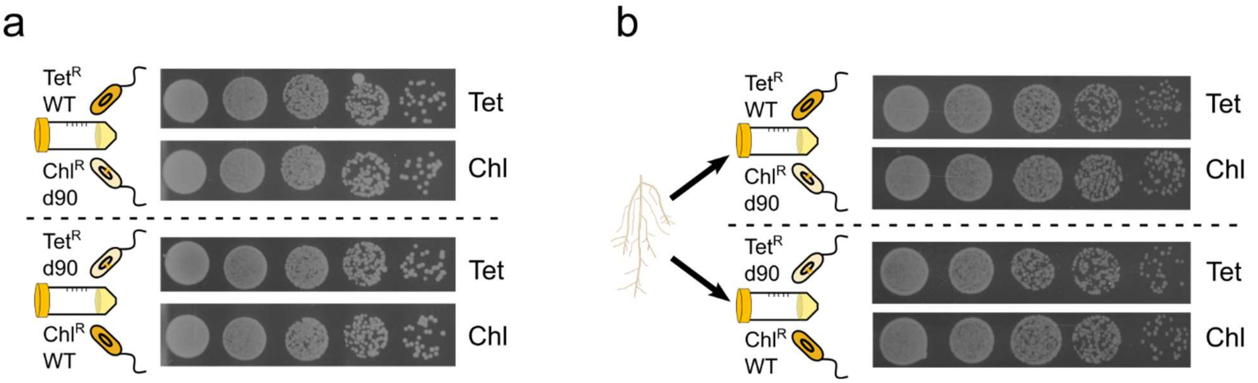

A Chloramphenicol resistant (ChlR) *Paraburkholderia bryophila* MF376 clone with scarless deletion of the shoot association efflux pump (d90, light orange) was competed against a Tetracycline (TetR) resistant wildtype clone (WT, dark orange) overnight in growth medium without plant extract (**a**) or with 2:1 dilution of *A. thaliana* Col-0 root extract in medium (**b**). Bacterial growth was quantified by plating serial five-fold dilutions of 1:1000 diluted overnight culture (left to right) on agar plates containing differentiating antibiotics (left), demonstrated that, the wildtype and d90 mutant both grow well in medium and in root extract. A reciprocal experiment (bottom) confirmed these results.

Supplementary figure 5. Prevalence of *ef90* orthologs across bacteria from different habitats.

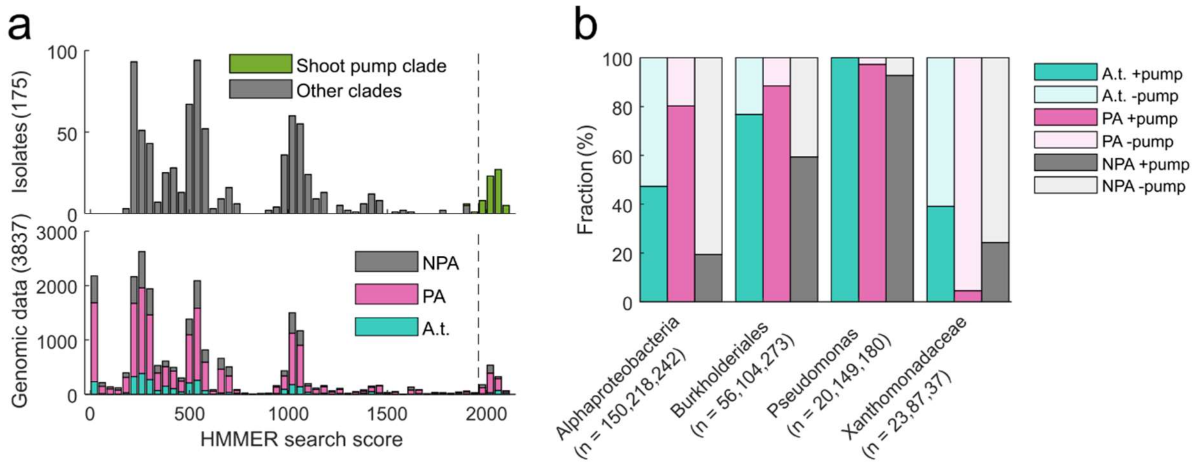

a, *ef90* homologs in the shoot-association clade are prevalent among bacteria from different habitats. A Hidden Markov Model (hmm) profile was built from members of the ef90 orthologous group using HMMER. (top) A histogram of the HHMER search score for the shoot clade hmm profile in the 175-member isolate collection derived largely from *Arabidopsis thaliana*. A strict HMMER threshold score (dashed line) includes the vast majority of genes in the shoot-association clade (*ef90 pumps*, green) and no other efflux pump genes (gray). (bottom) The HMMER search scores among genes in a large genomic database of 3837 bacterial genomes of Arabidopsis-associated (A.t., turquoise), non-Arabidopsis-plant-associated (PA, magenta), and non-plant associated (NPA, gray) bacteria. Genes in the shoot clade are right of the dashed line according to the HMMER threshold score found in the top panel. **b,** The prevalence of genomes that have a gene in the shoot-association clade in four Pseudomonadota taxonomic groups. Genomes that have at least one gene in the shoot-association clade of efflux pumps (+*ef90* pumps, dark color) are more prevalent among Arabidopsis-associated bacteria (A.t., turquoise) than among non-plant associated (NPA, gray) bacteria in all four taxonomic groups tested. Compared to no-Arabidopsis-plant-associated bacteria (PA, magenta), *ef90* orthologs are either more (Pseudomonas and Xanthomonadaceae) or less (Alphaproteobacteria and Bukholderiales) prevalent among Arabidopsis-associated bacteria.

Supplementary figure 6. The *saxF* efflux system from *Pseudomonas syringae* pv. tomato DC3000 is a member of the *ef90* orthologous group.

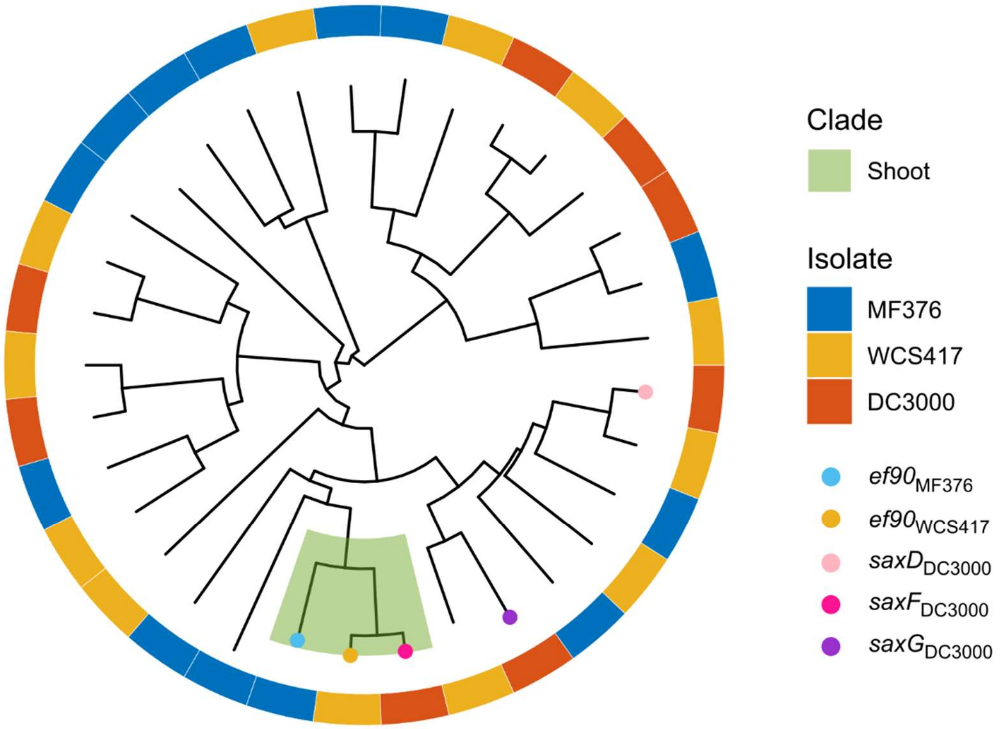

A gene tree of all inner membrane subunit RND-type efflux pumps from *Paraburkholderia bryophila* MF376, *Pseudomonas simiae* WCS417, and *Pseudomonas syringae* pv. *tomato* DC3000. The *saxF* gene from DC3000 sits within the shoot association clade defined by the MRCA of the ef90 orthologs from MF376 and WCS417.

Supplementary figure 7. The *ef90* ortholog in *Pseudomonas simiae* WCS417 does not confer resistance to shoot extract of either *Capsella bursa* or *Arabidopsis thaliana* Ler-1

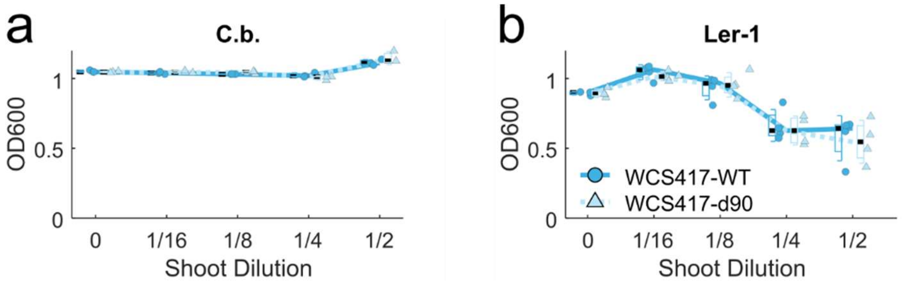

Fitness of *Pseudomonas simiae* WC417 d90 in macerated leaf extract. Final OD of overnight culture of wildtype *Pseudomonas simiae* WCS417 (WCS417-WT, dark cyan, solid line) or the mutant in the homologous shoot association efflux pump (WCS417-d90, light cyan, dashed line) in medium supplemented with diluted macerated leaf extract from *Capsella bursa* (**a**) or *Arabidopsis thaliana* Ler-1 (**b**). The mutant's growth inhibition seen for *Arabidopsis thaliana* Col-0 (Fig. 3d) is not seen for either host.

Supplementary figure 8. Expression *ef90/saxF* is induced by shoot extract from *Arabidopsis thaliana* Col-0 but not by *Capsella bursa-pastoris*

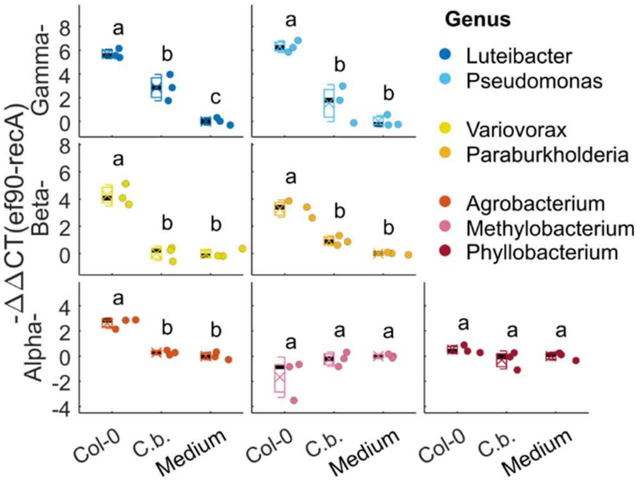

The expression of the ef90 pump of representative strains was measured using RT-qPCR 15 minutes after exposure to stimulus (n=3 independent cultures). The ef90 pump is induced to higher levels than by C.b. by five of seven representative strains (Fig. 5e) from all classes (Anova with post-hoc Tukey HSD test, P-value<0.05)

Supplementary figure 9. The diverse bacterial inhibitory profile of *Arabidopsis thaliana* genotypes

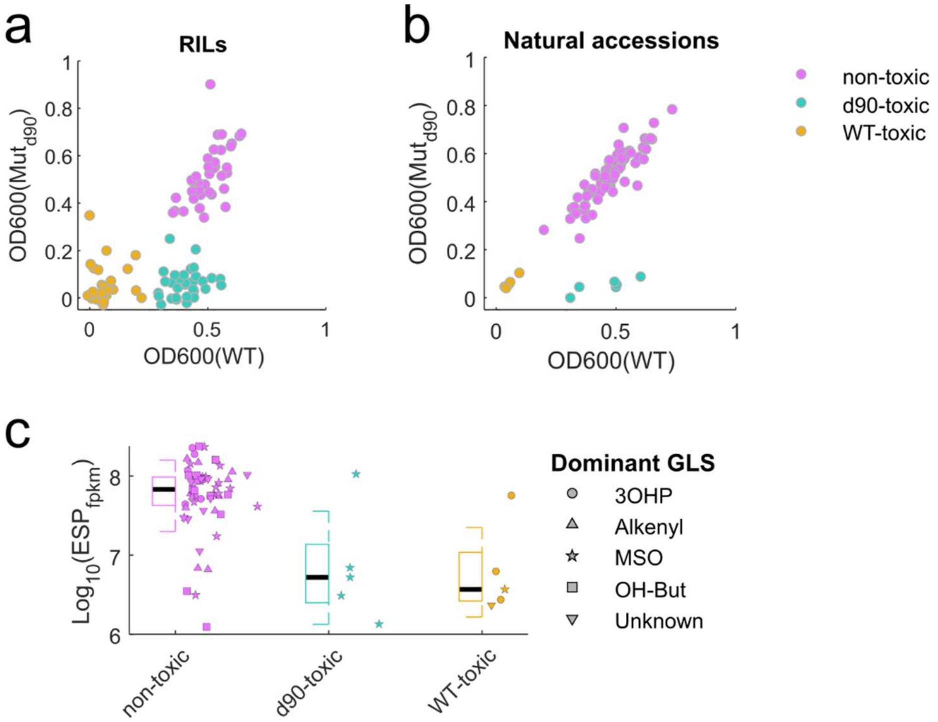

Final optical density of wildtype (WT) and the mutant d90 of Paraburkholderia bryophila MF376 after an overnight growth with a 2:1 mix of 2XYT medium and leaf extract of Col-0xLer-0 RILs (**a**) or natural accessions (**b**). In both populations we identify three groups with distinct inhibition profile: non-toxic, don't inhibit bacterial growth (magenta); d90-toxic, inhibit only the growth of the d90 mutant and not of the WT bacteria (turquoise); WT-toxic, inhibit the growth of both mutant and WT bacteria (yellow). **c,** The three phenotypic groups diverge in ESP expression and dominant glucosinolate chemotype. The expression level of ESP and the dominant chemotype of natural accession divided by phenotypic group. The expression of ESP is higher among non-toxic accession compared to both toxic groups. Looking at the dominant glucosinolate type we show that all d90-toxic accessions have MSO-GLS and three of the known four WT-toxic accessions have 3OHP-GLS, both in agreement with Fig. 6c. On the other hand, only one of the six non-toxic accessions that express low levels of ESP have either of them (the one outlier is accession 7378, Uk-1, with 4MSO-GLS).

Supplementary figure 10. The production of aliphatic glucosinolates is required for full inhibition of d90 growth

a b

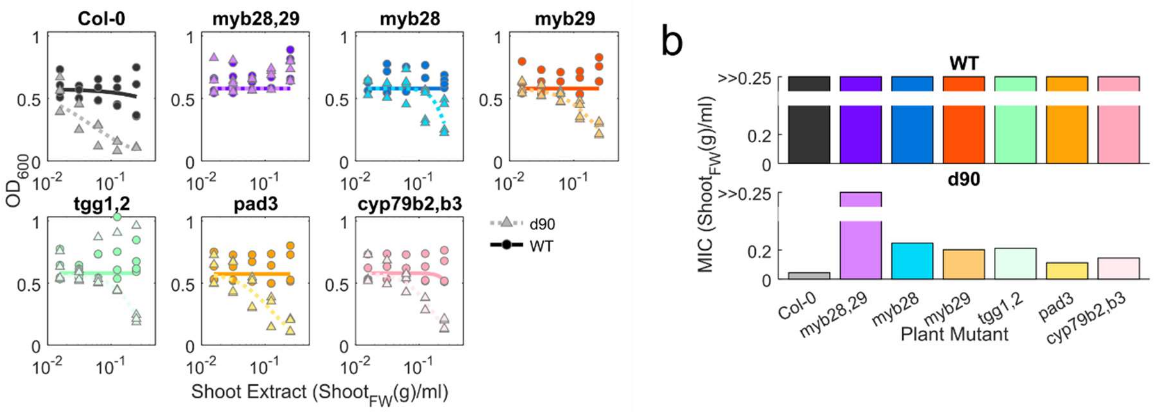

c

Name	Pathway	Effect
myb28,29	Aliphatic glucosinolates	Full loss of aliphatic glucosinolates
myb28	Aliphatic glucosinolates	Partial loss of aliphatic glucosinolates
myb29	Aliphatic glucosinolates	Partial loss of aliphatic glucosinolates
tgg1,2	Glucosinolates	Impaired myrosinase, slow GLS breakdown
pad3	Camalexin	Full loss of Camalexin
cyp79b2,b3	Indole glucosinolates	Full loss of indole glucosinolates

a, Dose-response curves showing optical density of wild-type (WT, black circles) and *d90* mutant (gray triangles) *Paraburkholderia bryophila* MF376 strains after overnight incubation with varying concentrations of shoot extracts from wild-type *A. thaliana* Col-0 and six phytoalexin biosynthesis mutants. Minimum inhibitory concentrations (MICs) for WT (solid line) and *d90* mutant (dashed line) were calculated as the extract concentration inhibiting growth by 50% (IC50) based on Hill equation fitting. **b,** Comparative MIC values of wild-type Col-0 and six phytoalexin biosynthesis mutant extracts against WT (top) and *d90* mutant (bottom) Paraburkholderia bryophila MF376. Complete elimination of aliphatic glucosinolates (*myb28,29* double mutant) fully restored *d90* mutant bacterial growth. **c,** Summary of plant mutant genotypes examined in this study and their affected phytoalexin pathways.
